# Modern contraceptive utilization among female ART attendees in health facilities of Gimbie town, West Ethiopia

**DOI:** 10.1186/1742-4755-11-30

**Published:** 2014-04-14

**Authors:** Addisu Polisi, Ewenat Gebrehanna, Gezahegn Tesfaye, Fekede Asefa

**Affiliations:** 1 Department of ART, Filtu Zonal Hospital, Somali region, Ethiopia; 2 Department of Reproductive Health, Addis Continental Institute of Public Health, Addis Ababa, Ethiopia; 3 Department of Public Health, College of Health and Medical Sciences, Haramaya University, Harar, Ethiopia

**Keywords:** ART attendees, Contraceptive utilization, Family planning

## Abstract

**Background:**

In many areas of the world where HIV prevalence is high, rates of unintended pregnancy have also been shown to be high. Of all pregnancies worldwide in 2008, 41% were reported as unintended and approximately 50% of these ended in abortion. To address these problems family planning is the best solution. Therefore, the purpose of the study was to assess modern contraceptive use among females on ART in health facilities of Gimbie town, Western Ethiopia.

**Methods:**

A facility based cross-sectional study was conducted in Gimbie town, western Ethiopia from December 2012 to January 2013. HIV infected women of reproductive age group (15-49 years) who came for ART care follow up during the data collection period were included in the study. Data was collected using an interviewer administered questionnaire. Binary logistic regression and multivariate analysis were employed using SPSS version 17.

**Results:**

Three hundred ninety five women on ART have participated in the study. More than half, 224 (56.7%), of the respondents were using modern contraceptive, of whom 67 (30%) use dual contraceptive method. Having information on modern contraception is positively associated with modern contraceptive use with (AOR=6.3, 95% CI (1.67, 24.1)) and respondents who have family size ≤4 were 50% less contraceptive users than those who have family size >4 (AOR=0.51, 95% CI (0.27, 0.96)).

**Conclusion:**

In this study contraceptive use among HIV positive women is better than the general population. However, use of dual methods, long acting and permanent method of contraceptives were found to be low. Continuous and targeted information provision on modern contraceptive should be done.

## Background

HIV/AIDS continues to have disastrous medical, economic, social, and physical impacts on individuals, their communities and the nations of the world [1]. Sub-Saharan Africa is at the epicenter of the epidemic and continues to carry the full brunt of its health and socioeconomic impact. And, Ethiopia is among the countries most affected by the HIV epidemic [2]. Despite that many people living with HIV in the country, ART enables them a return to normal life including a resumption of sexual activity and desire for children. Unless appropriate care taken in sexual activity and desire to have children, it also means for HIV infected women that the chances of transmitting the infection to their children and to their partner are higher considering the high population momentum. Therefore, one pillars of the WHO global effort to prevent mother-to-child HIV transmission (MTCT) is the prevention of unwanted pregnancies in HIV-infected women [3].

In many areas of the world where HIV prevalence is high, rates of unintended pregnancy and unsafe abortion have also been shown to be high. Of all pregnancies worldwide in 2008, 41% were reported as unintended or unplanned and approximately 50% of these ended in abortion [4]. To address these problems family planning is the best solution. It helps HIV positive women to prevent unintended pregnancy, avoid stress of pregnancy and also plan desired pregnancy while minimization of transmission risk. However, little has been known about the utilization of modern contraceptive among HIV positive women under antiretroviral treatment. This study was therefore, aimed to find out contraceptive utilization among HIV positive women on ART in health facilities of Gimbie town.

## Methods and materials

The study was conducted in West Ethiopia, Gimbie town which is located at 410 km away from Addis Ababa towards the west. A facility based cross-sectional study was conducted from December 2012 to January 2013. HIV infected women of reproductive age group (15-49 years) who came for follow up at ART clinics during the data collection period and who started ART, had at least one visit to the selected health facility were included in the study. Clients who are known to be infertile, pregnant mothers, have hearing problem and mentally ill or critically ill were excluded from the study.

Sample size was calculated using open Epi version 2.3 by taking proportions of HIV positive women who use contraceptive (p = 0.46) [5], 95% confidence interval, maximum of 5% margin of error and adding 10% non-response rate which gives a total sample of 424. The data was collected from two hospitals and one health center found in the town. The sample required for this study was proportionally allocated to each of these facilities based on number females on ART. Systematic random sampling method was employed to select participants in each of the facilities by using client flow as sampling frame.

A structured interviewer administered questionnaire was used for collecting the data which was prepared in English then translated to the local language Afan Oromo. Four diploma nurse data collectors and two BSC nurse supervisors were involved in data collection after trained on the purpose of the study, on how to make the interview, how to complete the questionnaire and ethical issues. The principal investigator and supervisor made day to day onsite supervision during the time of data collection.

Current use of modern contraceptive (use of any form of modern contraceptive by ART attendee women to delay or avoid pregnancy in the past 30 days) is the dependent variable. And, the independent variables include demographic characteristics (age, marital status, religion, educational status), reproductive factors (number of children, number of pregnancy, desired family size, intention to have another child), social factors (decision making on RH, occupation, income), clinical factors (Duration on ART, ART status), and service-related factors.

The collected data were entered, edited and cleaned using Epi info version 3.5.1 and analyzed using SPSS version 17. Statistical tests such as chi-square, measures of association (odds ratio (OR) with 95% confidence interval (CI) and P value) were calculated. The factors with P ≤ 0.2 values in bivariate analysis were retained for multivariate analyses. Adjusted odds ratios (AOR) with 95% confidence intervals (CI) and P <0.05 were considered statistically significant.

Ethical clearance was obtained from University of Gondar research ethics committee and Addis Continental Institute of Public Health (ACIPH) and Oromia regional Health bureau. Informed consent was obtained from each study subjects before conducting the actual interview. The confidentiality and privacy of the study participants were strictly maintained.

## Results

### Socio-demographic and reproductive health related characteristics

A total of 395 women agreed to participate in the study making the response rate 93.2%. The rest, 29 (6.8%), refuse to participate in the study. The mean age of study participants was 32.35 years (SD ±5.964 years) & 215 (54.4%), of the study participants were found in the age group of 25-34 years. One hundred forty nine, 149 (37.7%), of the respondents were protestant and 148 (37.5%), were Orthodox Christian. Majority of the respondents, 294 (74.4%), were married and 277 (70.1%) attended formal education. More than one third, 138 (34.9%), of the study participants has no means of income (Table 1).

**Table 1:** Socio-demographic and reproductive health related characteristics of female ART attendees in health facilities of Gimbie town, West Ethiopia, 2013

**Variables**	**Frequency**	**Percent**
**Age (years)**		
15-24	41	10.4
25-34	215	54.4
≥35	139	35.2
**Marital status**		
Never married	16	4.1
Married	294	74.4
Others*	85	21.5
**Religion**		
Protestant	149	37.7
Orthodox Christian	148	37.5
Muslim	38	9.6
Adventist	35	8.9
Catholic	22	5.6
**Educational status**		
No school	118	29.9
Primary school	134	33.9
Secondary school or above	143	36.2
**Income (per month)**		
No income	138	34.9
≤500 ETB (≤27 USD)	137	34.7
500-1500 ETB (27-81 USD)	109	27.6
≥1500 ETB (≥81 USD)	11	2.8
**Ever pregnant (n = 395)**		
Yes	338	85.6
No	57	14.4
**Total number of pregnancy (n = 395)**		
None	57	14.4
1-2	157	39.7
3-4	131	33.2
≥5	50	12.7
**Number of live births (338)**		
None	76	22.5
1-2	97	28.7
3-4	115	34
≥5	50	14.7
**History of still birth (n = 338)**		
Yes	37	10.9
No	301	89.1
**History of abortion (n = 338)**		
Yes	34	10.1
No	304	89.9
**Intention to have another child ever in the future (381)**		
Yes	98	25.7
No	283	74.3
**Desired number of children in addition to existing (n = 98)**		
1	47	48
≥2	51	52
**Family size (n = 364)**		
≤4	188	51.6
>4	176	48.4

*Others: such as widowed, separated and co-habiting.

The mean age at first sexual intercourse was 17.1 years (SD = 2.28). Among 395 respondents majority 338 (85.6%) of the women had reported a history of pregnancy at least once in their life time. Of these, 37 (10.9%) and 34 (10.1%) pregnancies were ended with still birth and abortions respectively. Among those 98 (25.7%) women who expressed their desire for number of children in the future, 47 (48%) and 51 (52%) had wished to have 1 & ≥ 2 children respectively. Concerning the family size, 188 (51.6%) of the respondents wants to have a family size of ≤4 and the rest, 176 (48.4%) wants to have a family size which is >4 (Table 1).

### Information on contraceptive and current use

Among 395 study participants, majority 364 (92.2%) of them have mentioned hearing about at least one type of modern contraceptive from different source of information. The highest percentage of the participants 332 (91.2%) heard from health professional followed by TV 103 (28.3%). Concerning information about specific method of modern contraception 279 (76.7%) pill, 271 (74.5%) injectable, 290 (79.7%) condom were the most commonly known types of contraceptives.

This study showed that 224 (56.7%) of the respondent were using modern contraceptive in the past one month prior to the survey. With regards to the specific contraceptives, pills 20 (8.9%), injectables 25 (11.2%), IUCD 6 (2.7%), implants 21 (9.4%), condom 77 (34.4%), vasectomy 3 (1.3%) and female sterilization 5 (2.2%), and 67 (30%) use dual methods of contraceptives. Among the current users, 54 (24.1%) want to have more children and 170 (75.9%) do not want to have more children. While among the nonusers, 50 (29.2%) want to have more children and 121 (70.8%) do not want to have more children.

The number of respondents ever used modern contraceptive was 336 (85.1%). The non users reported that they are not using modern contraceptive due to fear of complication with ART drugs 72 (42.1%) followed by 45 (26.3%) fertility related reasons, 12 (7%) partner opposition to use, 22 (12.9%) method related reasons and 15 (8.8%) want to have a child (Table 2).

**Table 2:** Information on modern contraceptive and current contraceptive use among female ART attendees in health facilities of Gimbie town, West Ethiopia, 2013

**Variable**	**Frequency**	**Percent**
Ever heard about modern	364	92.2
Contraceptive		
**Source of information**		
Health professional	332	91.2
TV	103	28.3
Radio	72	19.8
Friends	44	12.1
News paper and magazines	14	3.9
Community event	76	20.9
From pamphlets/ booklet/posters	13	3.6
**Methods respondent heard about**		
Pills	279	76.7
Injectables	271	74.5
IUCD	100	27.5
Implants	127	35
Condom	290	79.7
Vasectomy	60	16.5
Female sterilization	65	17.9
**User’s characteristics**		
**Ever used contraceptive method**		
Yes	336	85.1
No	59	14.9
**Current contraceptive users**		
Yes	224	56.7
No	171	43.3
**Reasons for not using contraceptive currently (n = 171)**		
Fear of complication with ART drugs	72	42.1
Fertility related reasons	45	26.3
Partner opposition to use	12	7
Method related reasons	22	12.9
Want to have child in the future	15	8.8
Others	5	2.9

One hundred fifteen (51.3%) of current users got contraceptives from ART clinic where they have HIV care and follow up, the rest got contraceptive from family planning clinic (unit) of the health facilities 59 (26.3%), NGOs health facility 27 (12.1%), Pharmacy 13 (5.8%), private clinic 2 (0.9%) and HEWs (Health Extension Workers) 8 (3.6%). Majority of the users preferred site of family planning service delivery was ART clinic (75%) where they usually go for HIV care and follow up. The majority 248 (62.8%) of the study subjects were on ART for greater than 12 months, 135 (344.2%) for less than 12 months and the rest 12 (3%) don’t remember. Respondent were also asked about their partner’s HIV-status in which majority 331 (83.8%) were concordant, 15(3.8%) were discordant and 49 (12.4%) missing.

### Husband’s knowledge and opinion on their wives use of contraceptive

Out of the total 224 current contraceptive users 213 (95.1%) of the study participant’s husband know the method their wives are currently using. Two hundred one (89.7%) of the husband attitude toward the use of modern contraceptive is supporting, 17 (7.6%) neutral and the rest 5 (2.2%) was against the use of modern contraceptive.

In this study it was found out that 67% of the decisions to use modern contraceptive and 56% to have a child were decided jointly with their husband. It is only 24% and 20% was decided by a woman to have a child and to use modern contraceptive respectively. The rest 13.4% of the decisions to use of contraceptive and 20% to have children were decided by the husband.

### Factors associated with use of contraceptive

Initially bivariate analysis was done to check for the crude association of different variables with contraceptive use. Two variables (marital status and information on modern contraception) were crudely associated with contraceptive use. On multivariate analysis, information on modern contraception have positive association with contraception use (AOR = 6.32, 95% CI (1.67, 24.07) and respondents who have family size ≤4 are 50% less contraceptive users than those who have family size >4 (AOR = 0.51, 95% CI (0.27, 0.96) (Table 3).

**Table 3:** Multivariate logistic analysis of factors associated with contraceptive utilization among female ART attendees in health facilities of Gimbie town, West Ethiopia, 2013

**Variable**	**Category**	**Yes**	**%**	**AOR (95% CI)**
Age	15-24	24	10.7	1.0
	25-34	118	52.7	2.84(0.96,8.45)
	≥35	82	36.6	2.41(0.77,7.55)
Marital status	Never married	6	2.7	1.0
	Married	192	85.7	0.35(0.06,2.16)
	Others*	26	11.6	1.86(0.28,12.46)
Educational status	No school	64	28.6	1.0
	Primary school	78	34.8	0.80(0.41,1.55)
	Secondary and	82	36.6	1.08(0.56,2.08)
	above			
Family size	≤4	116	55.5	**0.51(0.27,0.96)**
	>4	93	44.5	1.0
Income of respondent	No income	78	34.8	1.0
	≤27 USD	66	29.5	1.51(0.82,2.82)
	27-81 USD	71	31.7	0.81(0.41,1.60)
	≥81USD	9	4.0	0.56(0.17,2.99)
Number of children alive	None	76	22.3	2.43(0.85,6.97)
	1-2	97	28.7	0.63(0.23,1.78)
	3-4	115	34.0	0.66(0.28,1.54)
	≥5	50	14.7	1.0
Information on modern contraceptive	Yes	215	98.2	**6.32(1.67,24.07)**
	No	4	1.8	1.0

*Others: such as widowed, separated and co-habiting.

## Discussions

Our study showed contraceptive use among HIV positive women are better than the general population. But, the prevalence of dual methods, long acting and permanent method of contraceptives were found to be low. The main reasons for non uses of modern contraceptives were fear of contraception related complication with ART drugs followed by fertility related reasons. Most of the current contraceptive users preferred ART clinic for family planning service delivery. Information on modern contraception and family size were found to be associated with contraceptive use.

Majority, 332 (91.2%), of the study participants like that of study done in Southern Ethiopia [6] their main source of information was health professional. This might be due to the fact that communities perceives health worker as a true source of information about contraceptive compared with other sources [7]. Having information on the use of modern contraception was found to be significant predictor of contraception utilization. This finding was similar to another study [8].

This study revealed that, 336 (85.1%), of the respondent used at least one method of modern contraceptive method in the past. The current contraceptive utilization level was found to be 56.7%. This is inconsistent with different studies in Addis Ababa (73.3%) [9], Northern Ethiopia (46.3%) [5], Nekemte town (66.4%) [10] and South Africa (89%) [11]. This difference might be due to difference in socio-demographic and difference in study setting. On the other hand, a study done on general population shows the prevalence of contraception use to be 20% [12]. The possible explanation for this difference might be due to the knowledge of the study subjects are increased due to awareness creation by peer educator and health professionals.

More than half, 115 (51.3%), of current users got contraceptives from ART clinic where they have HIV care and follow up. But, three forth, (75%), of the current users preferred to get family planning service at ART clinic where they usually go for HIV care follow up. This is similar with studies done in Uganda [13] and Kenya [14] which majorities were preferred family planning service at ART clinic. This preference has a programmatic implication that the client need to get family planning service along with their HIV care and follow up thereby integrating HIV/AIDS chronic care with family planning services [13-16].

In this study, the utilization of contraceptive methods was dominated by short acting family planning methods. It was only, 21 (9.4%), of respondents who were found to use implants. This finding were consistent with the study done in Mekele town [7] which was 10.6%. And, IUCD users were found to be 6 (2.7%) which was more than the study done at Mekele (1.5%), and, even no IUCD use was reported in Cape Town [11]. This difference might be due to the fact that large number of the women had misconception about IUCD and its side effects such as interference with sexual intercourse, cancer and delays pregnancy.

Out of the, 224, current contraceptive users, 213 (95.1%), of the study participant’s husband/partner know the method their wives/partner are using. And, 201 (89.7%), of the husband/partners’ have supportive attitude towards the use of modern contraceptive. Similarly, positive effect of husband’s involvement on couple’s contraception use has been widely demonstrated in different studies [17-19].

In this study respondents who have family size ≤4 are 50% less contraceptive users than those who have family size >4. This difference might arise from perception that having more children is good in case if one child passed away the other will survive.

The limitation of this study was, it doesn’t used qualitative study design to explore the societal, cultural norms and values that shape the influence of women’s status on contraceptive use behavior and social desirability bias is likely.

## Conclusion

Although contraceptive use among HIV positive women is better than the general population the prevalence of dual methods, long acting and permanent method of contraceptives were found to be low. The main reasons for nonuse of modern contraceptives were fear that contraception may create complication with ART drugs followed by fertility related reasons. Most of the current contraceptive users preferred ART clinic for family planning service delivery. Information on modern contraception and family size were found to be associated with contraceptive use. Continuous and targeted information provision on modern contraceptive should be done in addition to integrating (strengthening the already integrated) family planning services with HIV services.

## Competing interests

The authors would like to declare that they have no any competing interest.

## Authors’ contributions

AP has conceived of the study, carried out the overall design and execution of the study, performed data collection and statistical analysis. EG has critically revised the design of the study, data collection techniques and helped the statistical analysis. GT and FA have participated in the revision of the paper and drafted the manuscript. All authors read and finally approved this manuscript for submission.
